# Breast Cancer Information Behaviours and Needs among Singapore Women: A Qualitative Study

**DOI:** 10.31557/APJCP.2021.22.6.1767

**Published:** 2021-06

**Authors:** Lavinia Lin, Wee Ling Koh, Qing Huang, Jeong Kyu Lee

**Affiliations:** 1 *Saw Swee Hock School of Public Health, National University of Singapore and National University Health System, Singapore. *; 2 *National University of Singapore, Singapore.*; 3 *Singapore Cancer Society, Singapore.*

**Keywords:** Breast cancer, communication, qualitative study, Asian, mammography screening

## Abstract

**Background:**

There is growing evidence on cancer communication and its impact on cancer-related health outcomes; however, little is known about how women gain access to and use breast cancer information in the multi-ethnic Asian context. This paper aimed to explore the breast cancer information acquisition behaviours and needs among Singapore women who attended a community-based health organisation for mammography screening.

**Methods, design and setting:**

Qualitative data were collected through semi-structured interviews with 37 racially diverse, aged 50 and above women, who have received mammography screening within the past two years. The interviews were conducted at either the Singapore Cancer Society Clinic or participant’s home.

**Results:**

Although cancer information scanning was more prevalent than information seeking (91.9% vs. 62.2%), those who purposively seek information exhibited a higher knowledge level of breast cancer. The most commonly cited sources for information scanning were friends, television and family, and for information seeking were the Internet, pamphlets from a healthcare organisation/ public authority, and healthcare providers. Singapore women were well-informed about the benefits of mammogram; however, specific knowledge, such as modifiable risk factors, reasons for different screening options and the trade-off between harm and benefit, was still lacking which led to confusion about screening.

**Conclusion:**

Breast cancer health educational materials should provide clear and balanced information to give women a more accurate or realistic expectation about mammography screening. Study findings provide important implications for breast cancer education and programs to move beyond simply raising awareness and craft specific informative messages addressing the needs of the target group.

## Introduction

Breast cancer is the most common cancer diagnosis and the leading cause of cancer deaths among Singapore women. Between 2014 and 2018, 11,232 new cases of breast cancer were diagnosed and more than 2,100 women died from the disease, accounting for 17.3% of cancer deaths among females (National Registry of Diseases Office, 2021). Although the age-standardized breast cancer incidence in Singapore is much lower than those in Western countries, it has the highest incidence in Asia with a rate of 70.7 per 100,000 (National Registry of Diseases Office, 2021). While it is a significant public health concern in the country, only one in three Singapore women had undergone regular mammography screening in the past two years according to the clinical recommendations (Loy et al., 2015). In response to the high breast cancer incidence and low screening rate, the Singapore Health Promotion Board (HPB) has established the Screen for Life - Breast Cancer Screening Programme to raise public awareness and promote early cancer detection, reducing the burden of breast cancer mortality and improving treatment outcomes. This nationwide programme provides mammography screening at subsidised rates to all women above the age of 50 (Health Promotion Board, 2020). However, barriers to regular mammography screening persist, which include low perceived susceptibility towards breast cancer, negative perceptions about mammogram, financial issues as well as social and cultural barriers (Seetoh et al., 2014; Lim et al., 2015b; Lim et al., 2015c; Malhotra et al., 2016; Wong et al., 2017).

Identifying factors that influence mammography screening is helpful to pinpoint a specific cause, but a more crucial step may be to have a broad view of how women access, gain and interpret their health information. A body of research has indicated that health information acquisition behaviours play a major role in enhancing health knowledge and shaping lifestyle decisions (Ramírez et al., 2013; Shneyderman et al., 2016; Wigfall and Friedman, 2016). In general, two information acquisition behaviours have been proposed in the line of health communication literature: information seeking and information scanning. Health information seeking refers to the ways in which individuals search for health information about their health, risks and illnesses (Nelissen et al., 2017). The usual preferred seeking sources are the Internet and health professionals (Barnes et al., 2017). Another typology is information scanning, which occurs when individuals take in desired information incidentally through the exposure to mediated and interpersonal sources (Nelissen et al., 2017). In the context of cancer communication, empirical studies have associated both information acquisition behaviours with breast cancer-related knowledge, awareness, preventive behaviours and screening decisions (Wigfall and Friedman, 2016; Ghazavi-Khorasgani et al., 2018). Factors such as attention to media and frequency of interpersonal communication have been reported to be positively associated with knowledge and risk perception of breast cancer among women (Lee et al., 2013; Lee and Ho, 2015). To further understand these findings, Lee et al built an integrated model explaining the underlying mechanisms. They found that the mammogram-related information acquisition from the media was positively associated with reflective integration of media health information, which in turn linked to behavioural attitudes and norms shaping an individual’s behavioural intentions of mammography use (Lee et al., 2016). 

Clearly, health information sources play a role in influencing women’s attitudes and beliefs about breast cancer. As most research was conducted in the Western countries, little is known about how women gain access to and use breast cancer information in the multi-ethnic Asian context. By utilising the unique demographic population of Singapore, this paper aimed to explore the breast cancer information acquisition behaviours and needs among Singapore women who attended a community-based health organisation for mammography screening.

## Materials and Methods

Qualitative data were collected in the form of semi-structured interviews from Singapore women aged 50 and above, who have received mammography screening within the past two years, specifically from July 2018 to June 2019 at the Singapore Cancer Society Clinic @ Bishan. The Singapore Cancer Society (SCS) is a community-based health organisation dedicated to minimising the impact of cancer in Singapore through the provision of public education, screening, patient services, financial assistance, research and advocacy (Singapore Cancer Society, 2020). SCS Clinic @ Bishan provides free mammography screening for female Singaporeans aged 50 years and above, holding a valid blue or orange Community Health Assist Scheme (CHAS) card, which indicates that their household monthly income per person was below 2000SGD (~1450USD) when receiving the mammography screening. This study was approved by the Institutional Review Board of the National University of Singapore (NUS). 

Using a purposive sampling technique, potential participants were identified from the SCS Clinic @ Bishan’s database based on important socio-demographic characteristics such as age and ethnicity (i.e. Chinese, Malay and Indian). From August 2019 to March 2020, a NUS research team member (LL) contacted potential participants via phone to explain the purpose and procedure of the study. Those who agreed to join the study were scheduled with an in-person interview at either the SCS Clinic^@^Bishan or their home. In total, 141 were contacted. Of which, 42 declined to take part, 61 were uncontactable, and one did not meet the criteria. To reduce nonresponses, multiple attempts through repeated calling and mobile messaging at different times of the day and weekends were employed. The data collection was halted due to the Covid-19 pandemic, and hence the projected sample size for Malay group was not reached (10 instead of 13 participants). Nevertheless, theoretical saturation was reached at a sample of 37, with a response rate of 26.2%. All participants provided written informed consent prior to the start of the interview. 

The interview guide was developed based on a literature review of women’s utilization of breast cancer screening services and health communication. It addressed topics of breast cancer screening practices and knowledge about mammography. It also consisted of open-ended questions assessing participants’ health communication channel and their health information needs. The interview was carried out in a language of the participant’s choice, and the duration ranged from 40 to 60 minutes. All but three interviews were completed in English; those that were conducted in non-English were translated into English. All interviews were audio-recorded and transcribed verbatim for analysis. Each participant received a 20SGD voucher in exchange for their time spent in the study. 

*Data analysis*

Two researchers (LL & WL) were involved in the data coding and analysis. Thematic analysis was conducted to allow themes to emerge from the data. Researchers familiarised with the interview data through multiple read-throughs of the transcripts to create an initial codebook. All transcripts were systematically coded and compared between researchers working independently to ensure coding consistency and establish inter-rater reliability. Any discrepancies in the coding were reviewed and discussed by all team members until consensus was reached. NVivo Pro Version 12 was used for the data analysis (QSR International Pty Ltd, 2018).

## Results

Table 1 shows the socio-demographic characteristics of the sample (N=37). Following a purposive sampling by ethnicity, our participants’ mean age was 59.1 years (SD=7.1) and 59.5% (n=22) had completed secondary education. About 70% (n=26) of them were currently married, with 81.1% (n=30) reported of having one or more children. 44.1% (n=15) were employed and 51.5% (n=17) reported to have a monthly household income below 2000SGD. A majority of participants seek primary healthcare from polyclinics (i.e. government public clinics) (83.8%; n=31) and had no family history of breast cancer (86.5%; n=32). Regarding breast cancer screening practices, about 90% (n=33) underwent regular mammography consistent with the Singapore breast cancer screening guidelines, and more than half (55.6%; n=20) practiced breast self-examination.

*Information search patterns and sources*

In this sample, cancer information scanning was more prevalent than information seeking (91.9% vs. 62.2%). For those with information seeking behaviours, some (24.3%) reported that they were only motivated to look for health information when their family members or they were in need of medical attention. 

Participant 1: *I never do any reading, like research on breast cancer. Not necessary at the moment. If I really need to, I will do the research. Now it’s not so urgent or a priority.*

Participant 11:* Unless certain things I’m concerned or relating to me or [my] family member, then I will google to find out more, read more.*

When comparing by ethnicity, educational levels and household income, no significant differences were found in information scanning behaviours. However, those with educational levels of primary school and below were less likely to actively seek health information (p=0.007). The most commonly cited sources for information scanning were friends, television and family; and for information seeking were the Internet, pamphlets from a healthcare organisation/ public authority, and healthcare providers. 

*1)Information scanning sources*

Participants were exposed to information about breast cancer or mammography screening through conversation with friends. Usually, the topic was initiated with friend’s health issues or suffering from breast cancer, which raised their awareness and perceived susceptibility to the disease. When hearing news about cancer diagnosis and treatment, they learned that breast cancer was particularly common among their age group. They also felt empathetic for their friends as the issue was highly relatable to their own health.

Participant 15: *How I know about it? Because so many of my friends had a breast cancer operation. When I see their operation, I don’t know painful or not painful. But I better get going to check. My friend, so many, two or three*.

Participant 14:* Recently, another friend of mine, she just had breast cancer. She just had her breast removed also. You know it’s so sad. She’ll put a towel here [over her breast].*

Interviewer: *To cover up?*

Participant 14: *Yes. Consciously, she’s doing it.*

From their friends, women also received recommendations and support to obtaining a mammography, which increased their intention to take charge of their own health.

Participant 30: *One of my friends went to Bishan clinic [i.e. SCS] for her mammogram regularly. And my friend advised me that it’s better to go for a check-up and not save these kind of money. I also told my older cousin but she said I don’t have to go for screening as it’s a very common issue. But I don’t want to listen to my cousin because it’s my own health and health is important.*

Local television (TV) news and advertisements were an important source of cancer information for the participants. For example, women came across free mammography screening events from the TV, which motivated them to have a check-up. TV has also served as an effective medium for education. A number of participants recounted viewing an old breast cancer awareness campaign in the past, promoting the importance of early detection and combating stigma around breast cancer. The ad campaign, which was available in all commonly used languages in Singapore, has made a lasting impact on their memory and recall.

Participant 12:* They had TV advertisements where they had all the four different languages. They would discuss about mammogram and everything. That was very much earlier, those days when they had this advertisement because people were very conscious. They’re a bit shy. At the time, it’s conservative to talk about mammogram and everything. I think a lot of people were not aware that there is such a thing that they had to go.*

Participant 16:* The government had been promoting on TV about the importance of mammogram, and it’s part of the health and wellness of your own being. I learn [from it] that it’s important to go for a check-up.*

Participant 26: *Some people don’t know the sign and symptom or where to go. Before on TV, they very frequent showing, showing, showing, but now no more already.*

*2) Information seeking sources*

Most frequently, participants reported searching the Internet for cancer information. They usually began their search process with Google, while some used social media sites, such as Facebook and YouTube. When asked whether they checked the credibility of the online information, some women made sure that they visited local health authoritative websites, for example the Health Promotion Board, Polyclinics and Singapore Cancer Society. However, others did not look at the web address and claimed that they had little knowledge on how to assess the credibility. 

Participant 28: *They claim to be doctors but not local because they are mostly foreigners. I don’t know whether they are real doctors or not but I just read, I don’t have these signs so I’m all right.*

Interviewer: *Is there any particular site that you would use?*

Participant 4: *No. As long as they put the shape of a breast, then I would read further.*

Participant 32: *I just Google anything based on breast cancer.*

Interviewer: *When you visit a particular site, do you check who runs or creates the website?*

Participant 32:* How to check I’m not sure.*

Pamphlets from a healthcare organisation/ public authority was another common source utilized by women when looking for cancer information. It was also their preferred resource for health information, followed by healthcare providers. Women referred that pamphlets were readily accessible and available from most public clinics, and covered the basic knowledge that they needed. They also commented that pamphlets were more reliable and trustworthy as these materials were developed by a trusted authority. 

Participant 23:* Information is there. In the clinic, there are booklets on the mammogram, on cervical cancer. I take the initiative to take the booklet back home, and read the signs and symptoms. That’s how you know that you’re having cancer and all that. *

Participant 3: *Brochure is printed by the hospital or by the Singapore Cancer Society. [So it] should not be false.*

*Information learnt*

Generally, participants revealed high levels of breast cancer awareness and acknowledged that early detection was the key to effectively managing the disease. As one participant put it, *“Cancer screening is to prevent; Prevention is better than cure.” *Our results found that different aspects of health information were obtained from different information-acquisition behaviours. Women who purposively seek information exhibited a higher level of specific knowledge about breast cancer and screening, such as risk factors, signs and symptoms of the disease, self-examination technique, and mammography screening guidelines. 

Interviewer: *What did you learn from the health brochure?*

Participant 30:* That I need to press my breasts and check if there are any lumps. Also we need to go for a check-up regularly for early detection. And health is important. *

Interviewer: *You mentioned that you read a lot about breast cancer from the Internet. Can you elaborate more about what you have learned?*

Participant 36: *Breast cancer, for one thing, I think it’s hereditary. Most of them say that it’s hereditary. If it’s in the family, chances of getting it is higher. There are times when they have to throw your whole breast away and you are breast-less. It’s good to be prepared. Then, the reason why they ask you to do all the exercises is to see whether there’s any lump or discharge, like blood or whatever. From what I know, having breast cancer doesn’t mean that you will die.*

For those who passively received information, they usually knew more about breast cancer health campaign-related information, for example free screening events and cancer survival stories. 

Interviewer: *How did you learn about mammogram?*

Participant 5: *I heard from news most of the time. Sometimes they give free mammograms. I feel it’s all right, if something is free and no harm, then I better go for the sake of my future and make sure I don’t get any breast cancer.*

Participant 29: *When I’m at home sometime, when I’m cooking, I listen to the radio. They were talking about, “This particular person is having breast cancer.” Then they talked about mammogram. *

*Information needs*

Most participants regarded the existing breast cancer information material to be adequate and easy to understand. To further advance their current knowledge, they have identified four important areas of breast cancer information needs: 

1) Prevention strategies - Participants were interested in the various risk factors, both genetic and lifestyle-related, as well as preventive methods for breast cancer. Women were aware that some established risk factors, such as age, reproductive history and family history, could increase one’s risk of developing breast cancer. However, they were uncertain about what lifestyle changes could reduce their chances.

Participant 9: *I don’t know how to prevent it if you have a family history. Very difficult to prevent. You can only maybe try to do regular screening. *

Participant 4: *Because it can be from internal, your internal condition or from because of your eating, your lifestyle. I don’t know what you have besides screening.*

2) Mammography results and the different screening options – Some women felt that the mammography reports were difficult to understand and “needed to be in more laymen terms.” As one participant explained that when she received her report, she did not know the meaning of ‘negative’ and thought “there’s something wrong.” Also, a majority of women did not know the differences between a mammogram and a breast ultrasound. They reported that both were used in detecting breast cancer, and some felt anxious when they were asked to go for both screening options.

Participant 9: *I don’t know why doctor sometimes give ultrasound, sometimes give mammogram. My confusion is why I must do ultrasound and mammogram within that year. The problem is because ultrasound is also x-ray and mammogram is also x-ray, that’s the period I start worrying.*

Participant 26:* Ultrasound has less pain and a mammogram has severe pain. Because of the pain they don’t want [to go for screening] and postpone until the things [cancer] spread. I was thinking instead of a mammogram, go for an ultrasound maybe can save more lives.*

3) Potential harms of overscreening – Although most women adhered to the current breast cancer screening guidelines, they expressed some concerns about the development of cancer in between the two-year surveillance interval and wished to go for annual screening. Without understanding the science behind the screening guidelines, they were mostly unware of the risks and harms of overscreening.

Participant 21: *Two years are too long. In between you might not know what could happen after one year then another one year to scan, and you find out there’s something. So I prefer one year.*

Participant 5: *I called and wanted to do it yearly but then the SCS told me that it must be two years. Two years are long, I am scared to see if anything happens to me.*

4) Access to affordable screening services – In our sample, all women underwent their mammography screening at the SCS Clinic^@^Bishan, which offers free cancer screening services to those with CHAS card. They said that the timely notifications sent by the Clinic via mail or phone were extremely helpful in reminding them about their upcoming mammography screening. All participants appreciated the efforts, and some suggested that the health authority in Singapore could follow the same approach to remind all eligible women to improve the mammography uptake. They also mentioned that affordable screening services should be emphasized in public health information material, as some were unaware of the availability of subsidised cancer screening services in public clinics. 

Participant 5:* Like where to have free screening, when do you get a discount or promotion. I would love to know.*

Interviewer: *Do you know that you could have your mammogram done in the Polyclinic?*

Participant 10:* No. Do they also do mammogram, Pap smear and everything? Do they have the machine?*

Interviewer: *Yes, they do provide cancer screening services in the polyclinic. *

**Table 1:** Socio-Demographic Characteristics and Breast Cancer Screening Practices of Participants (N=37)

Variables	N (%)
Age (Mean±SD)	59.1 ± 7.1
Ethnicity	
Chinese	13 (35.1)
Malay	10 (27.0)
Indian	13 (35.1)
Other	1 (2.7)
Education level	
Primary school or below	4 (10.8)
Secondary school	22 (59.5)
Diploma/ vocational certificate	6 (16.2)
University or above	5 (13.5)
Marital status	
Single	6 (16.2)
Married	26 (70.3)
Divorced/widowed	5 (13.5)
Number of children	
None	7 (18.9)
1 to 2	20 (54.1)
3 or more	10 (27.0)
Occupation*	
Employed	15 (44.1)
Homemaker	9 (26.5)
Retired	9 (26.5)
Unemployed	1 (2.9)
Monthly household income (SGD)*	
< 2000	17 (51.5)
2000 to 3999	11 (33.3)
> 4000	5 (15.2)
Primary care visits	
Private clinic	5 (13.5)
Polyclinic (i.e. Government public clinic)	31 (83.8)
Both	1 (2.7)
Breast cancer family history	
Yes	5 (13.5)
Grandmother	1 (2.7)
Mother	1 (2.7)
Sister	3 (8.1)
No	32 (86.5)
Breast self-examination practice*	
Yes	20 (55.6)
No	16 (44.4)
Mammography every two years	
Yes	33 (89.2)
No	4 (10.8)

*, Numbers may not add up due to missing values

## Discussion

This study aimed to explore breast cancer information acquisition behaviours and information needs among Singapore women who attended a community-based health organisation for mammography screening. In consistent with previous findings (Kelly et al., 2010; Leung et al., 2017; Nelissen et al., 2017), our study found that cancer information scanning was more prevalent than information seeking. Our results were also similar to those reported by Leung et al that the popular sources from where women received cancer information were TV and interpersonal network (Leung et al., 2017). For our participants, they did not only recall the detailed content of the TV breast cancer campaign but also maintained their behaviour changes. This suggests that repeated exposure to specific health information had a cumulative and lasting impact on behavioural choices (Hornik et al., 2013). Our results corroborate with previous findings that routine or opportunistic exposure to health content from non-medical sources plays an important role in promoting mammography uptake (Gollust et al., 2019). 

Prints from a healthcare organisation/ public authority and healthcare providers were among the most popular and preferred seeking sources of breast cancer information in this sample. This indicates that health professional sources remain the most highly trusted and reliable information to patients (Chua et al., 2018). Our results also showed a change in patterns of mediated sources used by Singapore older women, reporting an increasing trend in Internet utilization. With the vast available information online, the Internet is an increasingly popular source to feed patients’ health information needs (Chua et al., 2018; Zhang et al., 2020). Though it is readily available, our findings revealed that women lacked adequate skills to determine the credibility of online health information. As mentioned by one participant, she checked her symptoms using an online website unbeknownst to the source. This can be concerning, as it may lead to symptom misinterpretation and a delay in medical seeking (Lim et al., 2015a). Study findings point to the need of enhancing women’s online health literacy, and equipping them with tips to identify specific and high-quality credible online health information. 

Though scanning occurs more frequently than deliberate information seeking, our study found that women who purposively seek information exhibited a higher knowledge level of breast cancer and screening. For example, participants with seeking behaviours were able to explain in considerable detail about the risk factors and signs and symptoms of breast cancer, whereas with scanning behaviours were usually accompanied with information related to screening test and health campaign events. Aligned with the findings from Niederdeppe et al, the contexts of obtained cancer-related information are differed by information acquisition behaviours. The individual’s motivation, skills and capacities in health-information seeking enhance their specific knowledge, which may bring positive changes to their behaviour (Niederdeppe et al., 2007). Although health information scanning is less purposeful, screening test sites and health campaign events remain a good starting point for locating and engaging in authoritative health information.

In terms of breast cancer information needs, our participants identified four different areas that might require further clarification in the existing health educational material. These were: prevention strategies, mammography results and different screening options, potential harms of overscreening and access to affordable screening services. While many of the risk factors are not modifiable, some risk factors, such as obesity, use of oral contraceptives and alcohol consumption, can influence the occurrence of breast cancer (Guerrero et al., 2017; Nindrea et al., 2017). This information can be found on the official websites of the Health Promotion Board and Singapore Cancer Society, but it is not available in the printed material cited by our participants as their preferred health source. In addition, our results showed that some women may have misunderstandings and unrealistic expectations about mammography. It is noteworthy to mention that some of our participants preferred annual screening and were unaware of the risks and potential harms of overscreening. The confusion is understandable as a majority of health information offered by healthcare providers and printed material often overestimate the benefits of screening and underplay the potential harms that include overdiagnosis and negative psychosocial consequences (Gøtzsche et al., 2009; Hoffmann and Del Mar, 2017). Although mammography screening is effective at detecting breast cancer, the chance of having a false-positive result remains moderately high at 12% depending on age, risk factors and breast density (Nelson et al., 2016). Additional testing, such as diagnostic mammography, breast ultrasound, or breast biopsy, is thus needed to validate the abnormal finding on a mammogram. The unbalanced information may have misled and created wrong expectations about mammography where women view preventive screening programme as a tool to acquire feelings of security and reassurance that they are healthy (Østerø et al., 2014). Our finding highlights that a more comprehensive and balanced information is needed in the existing health material for the target audience, to provide them a realistic picture of the mammography screening and a better illustration of the screening procedure.

In comparison to the national’s screening rates, our study participants had an exceptional high adherence rate to the current clinical guidelines. This could be because of the free screening services coupled with timely mail or phone reminders provided by the SCS. Financial cost remains a barrier to regular screening mammography (Malhotra et al., 2016). To address this issue, the government has established the Screen for Life - Breast Cancer Screening Programme to encourage regular cancer screenings with a subsidised rate among Singaporeans (Health Promotion Board, 2020). However, our participants appeared to be unaware of the availability of cancer screening services in public clinics. More publicity of the national programme would be needed and should be emphasized in all public health educational material. Furthermore, screening reminders can be tailored with motivational messages and sent to women who are eligible for mammography screening at the national level. Meanwhile, other low-cost interventions, such as short message service reminders, might offer a sustainable solution for preventing missed appointments and therefore obtaining effective use of breast cancer screening in community (Uy et al., 2017). 

There are some limitations in the study. Participants were recruited from one community-based organisation, which may limit the generalizability of the study findings. Also, these participants receive regular reminders from the organisation; therefore, they may have a higher awareness about breast cancer and screening guidelines. Inherit recall bias may also present as interview subjects were asked with open-ended question about breast cancer information sources. It is possible that individuals with seeking behaviour have a higher recall rate than those with scanning behaviours, as the active search process may help them retain the information better than if it were come across incidentally. Nevertheless, this study provides insights into cancer information sources and needs through the lens of community-based screening participants in Singapore. The equal distribution of ethnicity also gives rise to a diversity of perspectives from a different racial or cultural background on the issue. 

In conclusion, cancer information scanning was more prevalent than information seeking among Singapore women, but those who purposively seek information exhibited a higher knowledge level of breast cancer. Results showed that Singapore women were well-informed about the benefits of mammogram; however, specific knowledge, such as modifiable risk factors, reasons for different screening options and the trade-off between harm and benefit, was still lacking. Furthermore, materials should provide clear and balanced information to give women a more accurate or realistic expectation about mammography screening. Study findings provide important implications for breast cancer education and programs to move beyond simply raising awareness and craft specific informative messages addressing the needs of the target group. 

## Author Contribution Statement

LJK and HQ conceptualized the study design. LL and KWL conducted interviews and performed data analysis. LL created the codebook and wrote the first draft of the manuscript. All authors took an active role in developing the original ideas for the study, participant recruitment, and critical revision of the manuscript for important intellectual content.

## Data Availability

All data relevant to the study are included in the article. Anonymised transcripts will be available upon request on a case-by-case basis.

## Approval

This study was approved by the National University of Singapore.
